# World Journal of Surgical Oncology reviewer acknowledgement 2012

**DOI:** 10.1186/1477-7819-11-28

**Published:** 2013-02-20

**Authors:** Manoj Pandey

**Affiliations:** 1 Institute of Medical Sciences, BHU, India

## Abstract

**Contributing reviewers:**

The editor of *World Journal of Surgical Oncology* would like to thank all our reviewers who have contributed to the journal in Volume 10 (2012).

## 

Daniel Abbott

United States of America

Ahmed Abdel Razek

Egypt

John Abraham

United States of America

Pedro Acién

Spain

David Adamson

United States of America

Lanre Adeyemo

Nigeria

Chalapathi Rao Adidam Venkata

Trinidad and Tobago

Gaurav Agarwal

India

MANISH Agarwal

India

Amit Agrawal

Nepal

Ferdinando Agresta

Italy

Hayam Aiad

Egypt

Takayuki Aimoto

Japan

Ilknur Ak

Turkey

Rita Alaggio

Italy

Andre Albergaria

Portugal

Luca Antonio Aldrighetti

Italy

Souhail Alouini

France

Ali Alzahrani

Saudi Arabia

Andrea Ambrosini-Spaltro

Italy

Luca Ampollini

Italy

Stylianos Apostolidis

Greece

Tomio Arai

Japan

Amira Arfaoui

Tunisia

Nikolaos Arkadopoulos

Greece

Henry Armah

United States of America

Miguel Angel Arrabal-Polo

Spain

Kunihiro Asanuma

Japan

Apichat Asavamongkolkul

Thailand

Stelios Assimakopoulos

Greece

Christos Asteriou

Greece

Maurice Efana Asuquo

Nigeria

M Hammad Ather

Pakistan

Goran Augustin

Croatia

Dagfinn Aune

United Kingdom

Abraham Ayantunde

United Kingdom

Umberto Baccarani

Italy

Flavia Baderca

Romania

Sasha Badzek

Croatia

Jong-Sup Bae

Korea, South

Xiaoyan Bai

China

Isabelle Bairati

Canada

Ravikanth Balaji

India

Aristotle Bamias

Greece

Maggi Banning

United Kingdom

Omar Barakat

United States of America

Dario Baratti

Italy

Nikolaos Barbetakis

Greece

Chris Barker

United States of America

Savio Barreto

India

Tommaso Bartalena

Italy

Bilal Battal

Turkey

Glenn Bauman

Canada

Alessandra Bearz

Italy

Marek Bebenek

Poland

Stephen Beck

United States of America

Thierry Bege

France

Rohit Behere

India

Kemal Behzatoglu

Turkey

Marcelo Beltran

Chile

Ehsen Ben Brahim

Tunisia

Frank Benedix

Germany

El Bachir Benjelloun

Morocco

Idriss Bennani-Baiti

Austria

Peter Benotti

United States of America

Adam Berger

United States of America

Martine Berliere

Belgium

Valentina Bertolini

Italy

Nikola Besic

Slovenia

Josko Bezic

Croatia

Paolo Pietro Bianchi

Italy

Eliana Bignotti

Italy

Karl Bilimoria

United States of America

Marcel Binnebösel

Germany

Saleh Binsaleh

Saudi Arabia

Ahitagni Biswas

India

Debabrata Biswas

United Kingdom

Almir Bitencourt

Brazil

Valerija Blazicevic

Croatia

Arye Blachar

Israel

Francesco Boccardo

Italy

Paolo Boffano

Italy

Sylvie Bonvalot

France

Viboon Boonsarngsuk

Thailand

David Boruta

United States of America

Jed Bouguila

Tunisia

Maria Brassesco

Brazil

Marko Brinar

Croatia

Giuseppe Brisinda

Italy

Hakan Brorson

Sweden

Andreas Brunner

Austria

Martin Buess

Switzerland

Mulazim Hussain Bukhari

Pakistan

Jean Butte

Chile

Aman Buzdar

United States of America

Giuseppe Cabibbo

Italy

Lu Cai

United States of America

Alpaslan Cakan

Turkey

Aras Emre Canda

Turkey

Matteo Angelo Cannizzaro

Italy

Dengfeng Cao

China

Giuseppe Cardillo

Italy

Giorgio Caretta

Italy

Giorgio Carrabba

Italy

Alfredo Carrato

Spain

Angelo Carretta

Italy

Pedro Carrión López

Spain

Jose Damián Carrillo-Ruiz

Mexico

Jorge Castillo

United States of America

Vincenzo Catalano

Italy

Fausto Catena

Italy

Abigail Caudle

United States of America

Miranda Chan

Hong Kong

Hong Chang

China

Kao-Ping Chang

Taiwan

Andrew Chang

United States of America

Wen-Cheng Chang

Taiwan

Huang Chang-Ming

China

Tushar Chattopadhyay

India

Paschalis Chatzipantelis

Greece

Yusheng Chen

China

George Chen

Hong Kong

Shuzhen Chen

United States of America

Xiaoping Chen

China

Fuxue Chen

China

Kaiyun Chen

China

Xian-Ming Chen

United States of America

Lin Chen

China

Yaw-Sen Chen

Taiwan

Michael Chen

Taiwan

Baoquan Cheng

China

Massimo Chiarugi

Italy

Kin-Fah Chin

Malaysia

Chintamani

India

Joanne Chionh

Australia

Seok-Reyol Choi

Korea, South

Siu Ho Kenneth Chok

China

Dean Chou

United States of America

Danai Chourmouzi

Greece

Terence Chua

Australia

Makoto Chuma

Japan

Byung Ha Chung

Korea, South

Jonas Cicenas

Switzerland

Saulius Cicenas

Lithuania

Tommaso Cioppa

Italy

Mark Clayer

Australia

Maria Elisabetta Coccia

Italy

Bertrand Coiffier

France

Tahsin Colak

Turkey

Alfredo Conti

Italy

Vito Domenico Corleto

Italy

Fabio Corsi

Italy

Renato Costi

Italy

Laura Crocetti

Italy

Laura Curiel

Canada

Giuseppe Curigliano

Italy

Vatsla Dadhwal

India

Zhijun Dai

China

Ichikawa Daisuke

Japan

Ronald Damhuis

Netherlands

Francesco D’Amico

Italy

Cem Dane

Turkey

Silvia Darb-Esfahani

Germany

Eelco De Bree

Greece

Remco De Bree

Netherlands

Germano De Cosmo

Italy

Bart De Keulenaer

Australia

Annelies Debucquoy

Belgium

Suha Deen

United Kingdom

Alberto Deganello

Italy

Andre Del Negro

Brazil

Paolo Del Rio

Italy

Silvia Dellapasqua

Italy

Hatice Mirac Binnaz Demirkan

Turkey

Adel Denewer

Egypt

Jing-Yu Deng

China

Didier Dequanter

Belgium

Marcello Deraco

Jordan

Christos Dervenis

Greece

Kristoffer Derwinger

Sweden

Pranab Dey

India

Isidoro Di Carlo

Italy

Luca Di Tommaso

Italy

Dolores Di Vizio

United States of America

Shiyong Diao

United States of America

David Diehl

United States of America

Daniel Dim

United States of America

Sean Dineen

United States of America

Sudeendra Doddi

United Kingdom

Mutlu Dogan

Turkey

Dietrich Doll

Germany

Yan Dong

United States of America

Pin Dong

China

Diego Dongiovanni

Italy

Dimitrios Dragoumis

Greece

Guillaume Ducarme

France

Prahlad Duggal

India

Padmaja Durga

India

Eleni Efremidou

Greece

Shigeru Ehara

Japan

Sigmund Ein

Canada

Magdy El Sherbiny

Egypt

Karim Elsahwi

United States of America

Ahmed Elshahat

Egypt

Shungo Endo

Japan

Gulgun Engin

Turkey

Joel Epstein

United States of America

Costantino Errani

Italy

Francisco Espin

Spain

Matthias Evert

Germany

Hong Fan

China

Luis Fariña

Spain

Maxime Fastrez

Belgium

Volker Fendrich

Germany

Qinghua Feng

United States of America

Luis Fernández Salazar

Spain

Jose Fernandez-Cebrian

Spain

Sergio Fernandez-Pello Montes

Spain

Mario Fernández-Ruiz

Spain

Gabriella Ferrandina

Italy

Ali Filiz

Turkey

J.E.F. Fitzgerald

United Kingdom

Aude Flechon

France

Irene Floriani

Italy

Patrick Forde

United States of America

Christina Fotopoulou

Germany

Amy Fowler

United States of America

Deliang Fu

China

Jianmin Fu

China

Teruhiko Fujii

Japan

Takeo Fujita

Japan

Takeo Fukagawa

Japan

Saburo Fukuda

Japan

Yosuke Fukunaga

Japan

Giuseppe Fusai

United Kingdom

Samia Gabal

Egypt

Giuseppe Gagliardi

Italy

Jessica Gahm

Sweden

Henning A Gaissert

United States of America

Trushar Gajjar

India

Georgios Gakis

Germany

Sean Galvin

Australia

Xiaohuan Gao

United States of America

Rajendra Singh Garbyal

India

Eduard Garc&Iacute;A-Cruz

Spain

Giuseppe Garcea

United Kingdom

Roberto Garcia-Navarrete

Mexico

Pankaj Kumar Garg

India

Sue Garnett

United Kingdom

Val Gebski

Australia

Roberta Gelmini

Italy

Stephanie Gentile

France

Venelin Gerganov

Germany

Suat Gezer

Turkey

Nandita Ghosal

India

Samer Ghosn

Lebanon

Yasemin Giles

Turkey

Luca Giovanella

Switzerland

Andrew Giustini

United States of America

Gabriel Glockzin

Germany

Rob Glynne-Jones

United Kingdom

Yih-Gang Goan

Taiwan

Ashish Goel

India

Ofer Gofrit

Israel

Henriette Golcher

Germany

Li Gong

China

Michel Gonzalez

Switzerland

Carmen Gonzalez

Spain

Rosario Granados

Spain

Filippo Grillo-Ruggieri

Italy

Viktor Grünwald

Germany

Jedrzejewski Grzegorz

Poland

Ivan Guerra

Spain

Dorothy Gujral

United Kingdom

Mehmet Ali Gulcelik

Turkey

Omer Gunal

Turkey

Serdar Gunaydin

Turkey

Wei Guo

China

Junming Guo

China

Cheng Guo

China

VISHAL GUPTA

India

Simona Gurzu

Romania

Niraj Gusani

United States of America

Tomonori Habuchi

Japan

Vijay Hadda

India

Yuni Elsa Hadisaputri

Japan

Nathan Hall

United States of America

Yukihiro Hama

Japan

Ken-Ichi Hanada

United States of America

Takeshi Hanagiri

Japan

Zhiming Hao

China

Noboru Hara

Japan

Kenichi Harada

Japan

Shuji Haraguchi

Japan

Julien Haroche

France

James Harrop

United States of America

Takahiro Hasebe

Japan

Yasuhisa Hasegawa

Japan

Yasushi Hashimoto

Japan

Ayshamgul Hasim

China

Stefan Hauser

Germany

Hermann Heimpel

Germany

Daniel Heise

Germany

Gisela Helms

Germany

Fernando Hernanz

Spain

Laszlo Herszenyi

Hungary

Stellan Hertegård

Sweden

Jun Hihara

Japan

Masahiko Hirota

Japan

Gabriella Honeth

Sweden

William Hope

United States of America

Chao-Wen Hsu

Taiwan

Gaosheng Huang

China

Jian Huang

China

Chung-Jen Huang

Taiwan

Chunling Huang

Australia

Shih-Ming Huang

Taiwan

Mina Hur

Kyrgyzstan

Wataru Ichikawa

Japan

Osamu Ikeda

Japan

Tomoaki Imai

Japan

Masahiko Inamori

Japan

Tsutomu Inaoka

Japan

Kashif Iqbal

Pakistan

Sherif Isaac

United Kingdom

Mitsuaki Ishida

Japan

Soichiro Ishihara

Japan

Yasuhiro Ito

Japan

Kazuo Itoh

Japan

Eyal Itshayek

Israel

Ognen Ivanovski

Macedonia

Lene Hjerrild Iversen

Denmark

Satoshi Iwasa

Japan

Teruo Iwasaki

Japan

Satoru Iwase

Japan

Takashi Iwata

Japan

Rakesh Jalali

India

Gayle Jameson

United States of America

Tae Jung Jang

Korea, South

Christian Jenssen

Germany

Han-Sin Jeong

Korea, South

Li Jian

China

Ming-Chung Jiang

Taiwan

Jun Jiang

China

Wei-Hong Jiang

China

Fevziye Kabukcuoglu

Turkey

Mitsutaka Kadokura

Japan

Witzel Kai

Germany

Hayato Kaida

Japan

Yoshihiro Kakeji

Japan

Toshiya Kamiyama

Japan

Tolga Kandogan

Turkey

Chang Moo Kang

Korea, South

Riken Karachi

Japan

Didem Karadibak

Turkey

Ikuko Kato

United States of America

Stamatis Katsenos

Greece

Jana Katuchova

Slovakia

Artur Katz

Brazil

Dara Kavanagh

Ireland

Hideki Kawai

Japan

Hideki Kawamura

Japan

Mehmet Kefeli

Turkey

Dezs Kelemen

Hungary

Venkata Kella

United States of America

Stephen Kennedy

United States of America

Barton Kenney

United States of America

Imran Khalid

Saudi Arabia

Suhail Aslam Khan

Ireland

Mohammad Khan

Pakistan

Joon Joon Khoo

Malaysia

Piya Kiatisevi

Thailand

Chumnan Kietpeerakool

Thailand

Takashi Kijima

Japan

John Kiluk

United States of America

Jonghan Kim

Korea, South

Namkyu Kim

Korea, South

Jong-Han Kim

Korea, South

Min-Chan Kim

Korea, South

Takahiro Kimura

Japan

Noriko Kimura

Japan

Olga Kiritsi

United Kingdom

Kentaro Kishi

Japan

Seigo Kitano

Japan

Safak Kiziltas

Turkey

Andreas Klein

Germany

Markus Klinger

Austria

Michiya Kobayashi

Japan

Hiroshi Kobayashi

Japan

Hirotoshi Kobayashi

Japan

Ercan Kocakoc

United States of America

Ercan Kocakoc

Turkey

Wachira Kochakarn

Thailand

Fumitaka Koga

Japan

Naoyuki Kohno

Japan

Yoshihiro Komatsu

United States of America

Katerina Kontogianni

Greece

Bon Seok Koo

Korea, South

Dimitris Korkolis

Greece

Athanassios Kotsinas

Greece

Gregory Kouraklis

Greece

Maria Koutourousiou

United States of America

Dimitrios Koutsimpelas

Germany

Friedrich Kreth

Germany

Andreas Krieg

Switzerland

Andreas Kroh

Germany

Haruki Kume

Japan

Chikara Kunisaki

Japan

Hiroki Kuniyasu

Japan

Eric C.H. Lai

Hong Kong

Laura Lambert

United States of America

Nirmal Lamichhane

Nepal

Antonios Lampidonis

Greece

Jeffrey Landercasper

United States of America

Hauke Lang

Germany

Stefan Langer

Germany

Stein Larsen

Norway

William Laskin

United States of America

Joseph W.Y. Lau

Hong Kong

Lester Layfield

United States of America

DANIELA LAZAR

Romania

Constantino Ledesma-Montes

Mexico

Yoon-Suk Lee

Korea, South

Jandee Lee

Korea, South

Seung-Jae Lee

Korea, South

Marcus Lehnhardt

Germany

Maryse Lemaire

Canada

Frank Lenze

Germany

Adam Levinson

United States of America

Miroslav Levy

Czech Republic

Stephen Lewis

United Kingdom

Wenqing Li

United States of America

Jordan Li

Australia

Guoli Li

China

Guojun Li

United States of America

Meixiang Li

China

Nguyen Liem

Viet Nam

Jen-Der Lin

Taiwan

Joerg Lindenmann

Austria

Julius Liptak

Canada

Caigang Liu

China

Ning Liu

China

Lorenzo Livi

Italy

Branimir Lodeta

Croatia

David Loeb

United States of America

Romaric Loffroy

France

Varut Lohsiriwat

Thailand

Visnu Lohsiriwat

Thailand

Ambrogio Pietro Londero

Italy

Jonathan Lopater

France

Roberto Lopes

Brazil

Francois Loubignac

France

Su-Ying Low

Singapore

Pei-Jung Lu

Taiwan

Shi-Ping Luh

Taiwan

Xueqing Lun

Canada

Andrea Luparia

Italy

Eric Lutz

United States of America

Huub Maas

Netherlands

Giulio Maccauro

Italy

Marcel Autran Cesar Machado

Brazil

Norman Machado

Oman

Andreas Machens

Germany

Thayur Madhusudhan

United Kingdom

Daichi Maeda

Japan

Antonio Maffuz

Mexico

Daruka Mahadevan

United States of America

Andreas H. Mahnken

Germany

Monica Malik

India

Giuseppe Malleo

Italy

Shramana Mandal

India

Mario Mandala

Italy

Ausilia Maria Manganoni

Italy

Eleftherios Mantonakis

Greece

Maurizio Marandola

Italy

Luigi Marano

Italy

Frédéric Marchal

France

Athanasios Marinis

Greece

Ugo Marone

Italy

Domenico Marotta

Italy

Giovanni Martinelli

Italy

Francesco Martines

Italy

David Martinez-Ramos

Spain

Miguel Martorell

Spain

Pavel Maruna

Czech Republic

Hitoshi Maruyama

Japan

Ibrahim Masoodi

Saudi Arabia

Hisahiro Matsubara

Japan

Masanori Matsuda

Japan

Astuji Matsuyama

Japan

Rajendra Prakash Maurya

India

Timothy Mccalmont

United States of America

Michael Mcfarlane

Jamaica

Sheu Meei-Ling

Taiwan

Asadi Mehrnaz

Iran

Giorgia Melli

Japan

Muhammed Ashraf Memon

Australia

Rudolf Mennigen

Germany

Roberto Merenda

Italy

Luca Messerini

Italy

George Metaxas

United Kingdom

Paolo Miccoli

Italy

Shuji Mikami

Japan

Robert C Miller

United States of America

Shinji Mine

Japan

Hiroyuki Mineta

Japan

Eric Mirallie

France

Reza Mirnezami

United Kingdom

Edita Miseikyte Kaubriene

Lithuania

Anuar Mitre

Brazil

Hiroshi Miyata

Japan

Yasuyoshi Miyata

Japan

Mona Mlika

Tunisia

Yasuhiko Mohri

Japan

Michele Molinari

Canada

Michael Montemurro

Switzerland

Basem Morcos

Jordan

Umberto Morelli

France

Marta Moreno Jimenez

Spain

Francisco J Morera-Ocon

Spain

Paolo Morgagni

Italy

Hiromasa Morikawa

Japan

Takuya Moriya

Japan

Satoru Motoyama

Japan

Charbel Moussallem

Lebanon

Barbara Mroczko

Poland

Pawel Mroczkowski

Germany

Sebastian Mueller

Germany

Oliver Mueller

Germany

Kristin Meisdalen Müller

Norway

Goo-Hyun Mun

Korea, South

Francisco Cristóbal Muñoz-Casares

Spain

Yoshiaki Murakami

Japan

Andrea Muratore

Italy

Paari Murugan

India

Toru Nagasaka

Japan

Koichi Nagata

Japan

Tomoyuki Nagata

Japan

Murlidharan Nair

United States of America

Itaru Naitoh

Japan

Keiichiro Nakamura

Japan

Hiroshige Nakamura

Japan

Ryoichi Nakanishi

Japan

Kozo Nakanishi

Japan

Yasuni Nakanuma

Japan

Atsushi Nambu

Japan

Vijay Naraynsingh

Trinidad and Tobago

Yoshitaka Narita

Japan

Peter Newcombe

Australia

Simon Ng

Hong Kong

Justin Nguyen

United States of America

Yasuhiro Nihon-Yanagi

Japan

Parvaneh Nikpour

Iran

Yuji Nimura

Japan

Toshirou Nishida

Japan

Kai Nowak

Germany

Kunihiko Numoto

Japan

Gerardo Andres Obeso Carillo

Spain

Toshiya Ochiai

Japan

Alexandre Odashiro

Canada

Hiroshi Ogawa

Japan

Temidayo Ogundiran

Nigeria

Adeyemi Ogunleye

United States of America

Jude Uzoma Ohaeri

Kuwait

Hidenori Ojima

Japan

Takehiro Okabayashi

Japan

Jiro Okami

Japan

Yukiyasu Okamura

Japan

Linus Ikechukwu Okeke

Nigeria

Masahide Oki

Japan

Tomohisa Okuma

Japan

Anabela Oliveira

Portugal

Mairin O’Mahony

Ireland

Ramesh Omranipour

Iran

Bert O’Neil

United States of America

Rok Orel

Slovenia

Antonio Orlacchio

Italy

Elena Orsenigo

Italy

Pablo Ortega-Deballon

France

Tomo Osako

Japan

Javier Osorio

Spain

David Otaegui

Spain

Gerd Otto

Gambia

S-H Ignatius Ou

United States of America

Tolutope Oyasiji

United States of America

Fabio Pagni

Italy

Sergio Renato Pais-Costa

Brazil

Sumanta Pal

United States of America

Weidong Pan

China

Chin-Chen Pan

Taiwan

Durgatosh Pandey

India

George Papacharalampous

Greece

Konstantinos Papaspyrou

Greece

Basilios Papaziogas

Greece

Tea Soon Park

United States of America

Heung-Woo Park

Korea, South

Jan Patino Mayer

Germany

Alberto Patriti

Italy

Subroto Paul

United States of America

Jasminka Pavelic

Croatia

Junjie Peng

China

Li Peng

China

Rodrigo Perez

Brazil

Jose Luis Perez Gracia

Spain

Marcos Perini

Brazil

Paolo Persichetti

Italy

Patrick Petignat

Switzerland

Francesco Petrella

Italy

Joachim Pfannschmidt

Germany

P. Terry Phang

Canada

Maria Picken

United States of America

Ymera Pignochino

Italy

Antonio Piñero

Spain

Marc D Piroth

Germany

Michele Pirrelli

Italy

Pompiliu Piso

Germany

Vassilis Pitsinis

United Kingdom

Benjamin Piura

Israel

Giorgio Pomara

Italy

Marco A Ponce-Camacho

Mexico

Lee Ponsky

United States of America

Jose Pontes Junior

Brazil

Maurilio Ponzoni

Italy

Camillo Porta

Italy

Konstantinos Potaris

Greece

Biju Pottakkat

India

Stephen P. Povoski

United States of America

Chris Protzel

Germany

Fabio Puglisi

Italy

Nicolai Purcz

Germany

Li Quan

China

Pradeep R

India

Vafa Rahimi-Movaghar

Iran

Marius Raica

Romania

Shahzad Raja

United Kingdom

Alexander Rapidis

Greece

Esther Raskopf

Germany

Sridhar Rathinam

United Kingdom

Federico Rea

Italy

Matthias Reeh

Germany

Leonardo Reis

Brazil

Bharat Rekhi

India

Zhenggang Ren

China

Marcelo Ribeiro

Brazil

Josep-Maria Ribera

Spain

James Richter

United States of America

Lorenza Rimassa

Italy

Yasushi Rino

Japan

Jan Lukas Robertus

Netherlands

Stuart Robinson

United Kingdom

Juan Pablo Rodrigo

Spain

Tania Rodrigues

Portugal

Simon Rogers

United Kingdom

Jong-Lyel Roh

Korea, South

Pantaleo Romanelli

Italy

Rey Romero-González

Mexico

Laszlo Romics

Ireland

Daphne Roos

Netherlands

Elisabeth Rooth

Sweden

Rafael Rosell

Spain

Robert Rosenberg

Switzerland

Danny Rosin

Israel

Marek Ruchala

Poland

Hannes A. Rudiger

Switzerland

Massimo Ruggieri

Italy

Fernando Ruiz Santiago

Spain

Alberto Ruol

Italy

Martin Rutegård

Sweden

Piotr Rutkowski

Poland

Primuharsa Putra S H A

Malaysia

Antonio Sa Cunha

France

Birch Saheeb

Nigeria

Takashi Saika

Japan

Kabul Saikia

India

Vasileios Sakellariou

United States of America

Hiroyuki Sakurai

Japan

Nobuyuki Sakurai

Japan

Nikolaos Salemis

Greece

Andrea Salmaggi

Italy

Milan Samardjiski

Macedonia

Alvaro Sanabria

Colombia

Luigi Santambrogio

Italy

Daniele Santini

Italy

Athanasios Saratzis

Greece

Aaron Sarver

United States of America

Akira Sasaki

Japan

Hidefumi Sasaki

Japan

Shuhei Sato

Japan

Sohei Satoi

Japan

Torill Sauer

Norway

Antonella Savio

Italy

Akira Sawaki

Japan

Christopher Schalchta

Canada

Orazio Schillaci

Italy

Jens Schittenhelm

Germany

Boris Schlenker

Germany

Hans J. Schlitt

Germany

Thomas Schmidt

Germany

Daniela Schmidt

Germany

Carl Schmidt

United States of America

Hans-Joachim Schmoll

Germany

Johann Schoenberger

Germany

Wolfgang Schroeder

Germany

Stephan Schwarz

Germany

Ilker Seckiner

Turkey

Andrada Seicean

Romania

Anuradha Sekaran

India

Serdar SEN

Turkey

Yasir Sepah

Pakistan

Ferdinand Serracino-Inglott

United Kingdom

Ramakrishnan Seshadri

India

Shimul Shah

United States Minor Outlying Islands

Omar Shah

India

Ashok Shaha

United States of America

Xiaobin Shang

China

Alok Sharma

India

B C Sharma

India

Shree Sharma

United States of America

Lizong Shen

China

Ming Shi

China

Daphne Shih Wen

Singapore

Kazuaki Shimada

Japan

Toru Shimazui

Japan

Mitsugi Shimoda

Japan

Takehiko Shimoyama

Japan

Dong Hoon Shin

Korea, South

Toshihiko Shinohara

Japan

Hisanori Shiomi

Japan

Norio Shiraishi

Japan

Kohei Shitara

Japan

Amal Shoma

Egypt

Rachna Shroff

United States of America

Parul Shukla

India

Neelaiah Siddaraju

India

Robert Siegel

Germany

Elcio Silva

Brazil

Giuseppe Simone

Italy

Michael Singer

United States of America

Shashideep Singhal

United States of America

Ingiridur Skirnisdottir

Sweden

Anne-Bine Skytte

Denmark

Louise Smart

United Kingdom

Russell Smith

United States of America

Yuji Soejima

Japan

Gemma Solon

Ireland

Christof M Sommer

Germany

Hong-Suk Song

Korea, South

Kyo Young Song

Korea, South

Petros Sountoulides

Greece

Caroline Speksnijder

Netherlands

Cosimo Sperti

Italy

Gian Paolo Spinelli

Italy

Parthi Srinivasan

United Kingdom

Eric Staals

Italy

Francesco Staffieri

Italy

Vladimir Stavrev

Bulgaria

Eberhard Stoeckle

France

Christine Stroh

Germany

Nathalie Sturm

France

Nazareno Suardi

Italy

Maher Sughayer

Jordan

Kenji Sugio

Japan

Iwao Sugitani

Japan

Tsuyoshi Sugiura

Japan

Michael Sugrue

Ireland

Dima Suki

United States of America

Yanan Sun

China

Valeriu Surlin

Romania

Tommaso Susini

Italy

Makoto Suzuki

Japan

Rei Suzuki

United States Minor Outlying Islands

Shuji Suzuki

Japan

Marian Svajdler

Slovakia

Carol Swallow

Canada

Mark Sywak

Australia

Karoly Szuhai

Netherlands

Marco Tafani

Italy

Panos Taflampas

United Kingdom

Tsuyoshi Tajima

Japan

Hiroki Takahashi

Japan

Yoshihisa Takahashi

Japan

Yuji Takakura

Japan

Kazuya Takamochi

Japan

Masashi Takano

Japan

Tadatoshi Takayama

Japan

Hiroyuki Takei

Japan

Hiroya Takeuchi

Japan

Hiroshi Takeyama

Japan

Hiromitsu Takeyama

Japan

Onder Tan

Turkey

Geok Chin Tan

Malaysia

Siao Pei Tan

United Kingdom

Kuniya Tanaka

Japan

Katsuhiro Tanaka

Japan

Nobumichi Tanaka

Japan

Kumiko Tanaka

Japan

Siriwan Tangjitgamol

Thailand

Tawesak Tanwandee

Thailand

Juan Tardío

Spain

Hirotaka Tashiro

Japan

Muhyittin Temiz

Turkey

Anthony Teoh

Hong Kong

Tadashi Terada

Japan

Koji Teramoto

Japan

Catherine Terret

France

Alessandro Testori

Italy

Oliver Thiele

Germany

Eric Thompson

United States of America

Guido Alberto Massimo Tiberio

Italy

Daniel Johannes Tilkorn

Germany

Marcos Tobias-Machado

Brazil

Giuseppe Toffoli

Italy

Osamu Tokuda

Japan

Jeffrey Tomaszewski

United States of America

Francesco Tonelli

Italy

Qiangsong Tong

China

Waraphan Toniti

Thailand

Takayuki Tooynaga

Japan

Guido Torzilli

Italy

Ryo Toya

Jamaica

Hidenori Toyoda

Japan

Trine Tramm

Denmark

Dragan Trivanovic

Croatia

Priti Trivedi

India

Pauline Truong

Canada

Gary Tse

Hong Kong

Nikolaos Tselis

Germany

Konstantinos Tsimogiannis

Greece

Alexander Tsivian

Israel

Hironori Tsujimoto

Japan

Kazunori Tsukuda

Japan

Toshio Tsuyuguchi

Japan

Shuiping Tu

China

Jean Jacques Tuech

France

Hagit Tulchinsky

Israel

Per-Ulf Tunn

Germany

Stavros Tyritzis

Greece

Shinichi Ueno

Japan

Hideki Ueno

Japan

Christoph Ulmer

Germany

Diclehan Unsal

Turkey

Andi Utama

Indonesia

Kenicni Utano

Japan

J. Fernando Val-Bernal

Spain

Ben Valdez

United States of America

Graciela Valero

Spain

Antonio Valezi

Brazil

Marcel Van De Poll

Netherlands

Ferdi Van Dorsten

Netherlands

Hein Van Poppel

Belgium

Kimberly Varker

United States of America

Jose Vartanian

Brazil

Vinicius Vazquez

Brazil

Sanjay Vikrant

India

Juan J. Vila

Spain

Gustavo Villalona

United States of America

Vincent Vinh-Hung

Belgium

Georges Vlastos

Switzerland

Christian Von Falck

Germany

Camelia Doina Vrabie

Romania

Kamil Vyslouzil

Czech Republic

Nobuyuki Wada

Japan

Alexander Wahba

Norway

Toshifumi Wakai

Japan

Zhou Wang

China

Zhong Wang

China

Daorong Wang

China

Jaw-Yuan Wang

Taiwan

Shulin Wang

China

Tracy Wang

United States of America

Shutao Wang

United States of America

Xiaoyan Wang

China

Irene Wapnir

United States of America

Oliver Warren

United Kingdom

Nir Wasserberg

Israel

Hidemichi Watari

Japan

Stefan Watzka

Austria

Michael Wayne

United States of America

Andreas Weber

Germany

Ulrich Wedding

Germany

Shu-Chen Wei

Taiwan

Huang Wen-Bin

China

Arne Westgaard

Norway

Christina Westhoff

Germany

Antonio Westphalen

United States of America

Theresa Whiteside

United States of America

Horace Williams

United Kingdom

Stefan Willis

Germany

Andrzej Wincewicz

Poland

Sze Chuen Cesar Wong

Hong Kong

Thian-Sze Wong

Hong Kong

Qiong Wu

China

CHIH-HSIUNG WU

Taiwan

Hongping Xia

Singapore

Huijuan Xiao

China

Li Xiaorong

China

Mingzhao Xing

United States of America

Rui-Hua Xu

China

Jianmin Xu

China

Takashi Yamada

Japan

Kazutaka Yamada

Japan

Hidehisa Yamadsa

Japan

Rin Yamaguchi

Japan

Masakazu Yamamoto

Japan

Manabu Yamamoto

Japan

Hidetaka Yamamoto

Japan

Yo-Ichi Yamashita

Japan

Ryo Yamashita

Japan

Lunan Yan

China

Zuozhang Yang

China

Qingwu Yang

China

Xueying Yang

China

Hideaki Yano

United Kingdom

Kazuhiro Yasuda

Japan

Lin Ye

United Kingdom

Qian Yeben

China

Mustafa Yilmaz

Turkey

Cheng-Har Yip

Malaysia

Yoshio Yoshida

Japan

Kazuhiro Yoshida

Japan

Takaki Yoshikawa

Japan

Tateki Yoshino

Japan

Hideyuki Yoshitomi

Japan

Gang Yu

China

Ohtsuki Yuji

Japan

Vuslat Yurut-Caloglu

Turkey

Evangelos Zacharakis

United Kingdom

S. Yousuf Zafar

United States of America

Menelaos Zafrakas

Greece

Michal Zamecnik, Jr.

Slovakia

Jason Zell

United States of America

Zhao-Chong Zeng

China

Kejun Zhang

China

Sen Zhang

China

Guoyou Zhang

Australia

Shiwu Zhang

China

Zhao

China

Shusen Zheng

China

Liduan Zheng

China

Yuan Zhongyu

China

M. Zhou

United States of America

Li Zhou

China

Jiang Zhu

China

Xianlu Zhuo

China

Richard Zigeuner

Austria

Luigi Zorcolo

Italy

Zvonimir Zore

Croatia

Debra Zynger

United States of America

